# Age, thyroglobulin levels and ATA risk stratification predict 10-year survival rate of differentiated thyroid cancer patients

**DOI:** 10.1371/journal.pone.0221298

**Published:** 2019-08-19

**Authors:** Antony Kelly, Bertrand Barres, Fabrice Kwiatkowski, Marie Batisse-Lignier, Bernadette Aubert, Clémence Valla, Frédéric Somda, Florent Cachin, Igor Tauveron, Salwan Maqdasy

**Affiliations:** 1 Service de médecine nucléaire, CLCC Jean Perrin, Clermont-Ferrand, France; 2 UMR INSERM 1240 "Molecular Imaging and Theranostic Strategy", Clermont Auvergne University, Clermont-Ferrand, France; 3 Département de recherche clinique, CLCC Jean Perrin, Clermont-Ferrand, France; 4 CHU Clermont-Ferrand, Service d’endocrinologie, diabétologie et maladies métaboliques, Clermont-Ferrand, France; 5 Laboratoire GReD: UMR Université Clermont Auvergne-CNRS 6293, INSERM U1103, BP, Aubiere, France; 6 Université Clermont Auvergne, Faculté de Médecine, Clermont-Ferrand, France; National Cancer Center, JAPAN

## Abstract

**Introduction:**

Differentiated thyroid cancer (DTC) is the most common of endocrine cancers. Many studies have focused on recurrence-free survival of DTC patients, however, few studies have addressed overall survival rates. Given its very good prognosis, estimating overall or long-term survival in patients with DTC seems rational. So far, neither the impact of pre- and post-ablation thyroglobulin, nor that of initial American Thyroid Association (ATA) risk stratification on long-term disease-specific survival, have been sufficiently studied.

**Objective:**

The aim of this study was to determine the factors that influence long-term disease-specific survival and thyroglobulin levels in patients with DTC who have been previously treated with thyroidectomy and radioactive iodine (RAI) remnant ablation.

**Patients and methods:**

This observational retrospective study included 1093 patients who were treated for DTC between 1995 and 2010 and are still monitored in our tertiary center. Only patients who needed RAI ablation after thyroidectomy were included in this study. Patients who were treated with RAI following rhTSH stimulation, patients who presented positive anti-thyroglobulin antibodies, and patients who had micro-cancers were excluded. Pre-ablation stimulated thyroglobulin (Pre-ablation sTg) was measured after thyroid hormone withdrawal (THW), just before RAI.

**Results:**

According to ATA standards, 29 patients (2.7%) were classified as high-risk patients. Initial ATA high-recurrence risk rating (HR 21.9; 95% CI: 8.5-56.3), age>55 years (HR 23.8; 95%-CI: 7.5-75.3) and pre-ablation sTg≥30 μg/l (HR 8.4; 95% CI: 4.6-15.3) significantly impacted ten-year survival. Moreover, age over 45 years, ATA moderate-risk and follicular DTC were also significant. Ten-year survival was lower in ATA high-risk patients (51% vs 95% and 93% for the low and intermediate risk; p<10^-7^), patients older than 55 years (82% *vs* 98%; p<10^-7^), and in patients with pre-ablation sTg≥30 (78% *vs* 95%; p<10^-7^). Three rates of long-term survival were distinguished: excellent (survival rate of 99% in patients<55 years with pre-ablation sTg <30μg/l) representing 59% of the cohort, moderate (survival rate of 94.5% in patients <55 years with pre-ablation sTg ≥30μg/l or ≥55 years with pre-ablation sTg <30 μg/l) representing 38% of the cohort, and low (survival rate of 49% in patients ≥55 years with pre-ablation sTg ≥30μg/l) representing 3% of the cohort.

**Conclusion:**

Initial ATA high-risk classification, age over 55 years old and pre-ablation sTg ≥30 μg/l are the main negative factors that influence the ten-year survival in DTC. We suggest three categories of overall survival rates. Patients older than 55 years with pre-ablation sTg ≥30 μg/l have the worst survival rate.

## Introduction

Differentiated thyroid cancer (DTC) accounts for 90% of all thyroid cancers [1]. It has an excellent prognosis, and long-term survival is sustained in the vast majority of the patients as a result of traditional treatment by surgery and radioactive iodine (RAI) [2]. Regular patient follow-ups are consequently adapted according to the risk of disease recurrence or persistence [3–5]. Most oncological studies have evaluated recurrence-free survival and long-term remission, however, due to its excellent prognosis, long-term survival is particularly interesting in DTC. Numerous studies have identified multiple factors correlated to overall survival rates. Two particularly influential factors that impact overall survival are RAI and total thyroidectomy [6,7]. Furthermore, age over 45 years old and male sex have been found to be significant factors that predict overall survival in patients with low-risk DTC, as well as TNM in poorly differentiated thyroid cancers [8–11]. However, studies evaluating long-term disease-specific survival have not focused on the role of thyroglobulin, or on that of the new risk stratification system suggested by the ATA in the prediction of the overall survival. To the best of our knowledge, the impact of pre- and post-ablation thyroglobulin and that of the initial ATA risk stratification on long-term disease-specific survival were rarely studied.

The aim of this study was to evaluate the predictive factors influencing ten-year disease-specific survival in patients with DTC.

## Patients and methods

We conducted an observational retrospective study to analyze clinical, biological and morphological data of patients treated for DTC (surgery and radioactive iodine) in our tertiary center between 1995 and 2010 that are still followed up.

### Database, inclusion and exclusion

In 1995, we established the “*Registre Thyroïde Auvergne*” clinical database in order to continuously update patients’ clinical, histological, biological and morphological data. Since then, 1500 patients who needed complementary radioactive iodine have been included in the register, with 7 to 15 year follow-ups. Micro cancers were excluded. Disease evolution, including cancer persistence, recurrence and localization, the surgical procedures and complementary treatments were regularly registered for each patient. TNM staging system of AJCC 2010 (7^th^ edition) was used. Thyroid capsule invasion (the difference between T2 and T3) and tumor size were taken into consideration in our cohort in order to be compatible with the 8^th^ TNM edition published just after the completion of the collection of our variables (2017).

In order to harmonize the management, patients with micro cancers, patients treated with RAI after rhTSH stimulation instead of thyroid hormone withdrawal (THW), patients with positive anti-thyroglobulin levels, and patients missing one of thyroglobulin measurements were excluded from the analyzed cohort.

### Ethical issues

Before analyzing our database, each patient was informed about the study by an individual information letter. In this letter, the aims of the study, the method of data collection and analysis were detailed. Each patient had a time interval to reply; any refusal of participation was respected. The ethics committee [Institutional Review Board:*”Comité d’Ethique des Centres d’Investigation Clinique (CECIC) d’Inter-région Rhône-Alpes-Auvergne”*, Grenoble, IRB 5921] approved the study on June 30, 2017.

### Thyroid cancer management

Total thyroidectomy was performed on all patients included in the study. DTC and its particularities (cell types), extension and staging were detailed by a pathologist for all patients. RAI ablation was administered to all patients included in the study.

Patients with cancer that was over 1 cm in diameter (T1b, T2, T3), with extra thyroidal extension (T3, T4), with lymph node involvement (N1a, N1b) or with distant metastases were treated with RAI. Patients presenting micro cancers of less than 1 cm diameter (T1aNxMx) were not treated by RAI and were therefore excluded from the cohort.

To ensure isotopic thyroid ablation, complementary RAI was administered after surgery under thyroid hormone withdrawal (THW). THW (LT4) was applied for five weeks before RAI. LT3 was administered for the first three weeks before being definitely stopped. Pre-ablation stimulated thyroglobulin (pre-ablation sTg) and anti-Tg antibodies were measured on the day of RAI therapy. A whole body scintiscan (WBS) with ^131^I was performed five days after RAI administration for diagnostic purposes. Measurement of rhTSH-stimulated serum Tg (Tg at first evaluation, or “Tg” in the manuscript) was performed six to nine months after RAI ablation. Cervical US and WBS were performed to evaluate the response to initial ablation. All patients were periodically evaluated by clinical, laboratory and ultrasonography tests. Additional CT scans at varying intervals were performed if deemed necessary during a multidisciplinary meeting.

### Thyroglobulin assays

Thyroglobulin measurement was performed in the laboratory of radio pharmacology in Jean Perrin Center, with a minimal detectable value of 0.1 μg/l. Immuno-radiometric assay with coated tubes (CisBio:TgIRMA) were used. The interference with antibodies was also evaluated.

### Risk stratification

Initial risk stratification was performed according to ATA recommendations. Low risk patients have intrathyroidal DTC, and nodal involvement limited to ≤5 micro metastases (<0.2 cm). A patient is judged to be at intermediate risk when an aggressive histology, minor extra thyroidal extension, vascular invasion or > 5 LN are present (0.2-3 cm). High risk patients have gross extra thyroidal extension, incomplete tumor resection, distant metastases or LN >3 cm [2].

### Evaluation of ten-year disease-specific survival

We limited the analysis to patients included between January 1995 and December 2010 in order to allow for a sufficient follow up period after initial treatment. Ten-year disease specific survival was defined as the time interval from the initial thyroid ablation at diagnosis to death. Surviving patients were censored at the last follow-up.

### Statistical analyses

The statistical analysis aimed first to describe the general characteristics of the population at inclusion. Quantitative parameters were expressed as means with standard deviation (median and range if distribution was not Gaussian), while qualitative parameters were described using population size and frequencies.

For all patients, pre-ablation sTg was measured under THW, while Tg at evaluation was measured under rhTSH stimulation. Thus, Tg study was robust with no bias related to stimulation method.

For the principal analysis, Student t-test, ANOVA or Kruskal-Wallis H-test (depending on normality and homoscedasticity of distributions) were used to identify the link between different qualitative and quantitative parameters and disease-specific survival. Pearson’s correlation or Spearman’s ranks correlation was used to test the relation between two quantitative variables. The prognostic value of different factors affecting the survival was estimated using the Kaplan-Meier method. Survival curve comparisons were achieved with Log-Rank and Mantel-Haenszel tests. The relative influence of several significant parameters was confirmed by a multivariate logistic regression analysis for dichotomous parameters and with the proportional hazard COX model for survival variables.

For secondary analyses, an analog of ROC analysis with exhaustive research of the optimal pre-ablation sTg cutoff was used to obtain the most discriminant cutoff for biological parameters. This cutoff was validated by Kaplan Meier analysis of overall survival.

All tests were two-sided and a standard p-value <0.05 was used as a significance threshold. SEM software was used for data-management and statistical calculations [12].

## Results

### Predictive factors of long-term disease-specific survival

The general characteristics of our cohort are summarized in **Table 1**. Women were predominant in the cohort. Papillary thyroid cancer was the most frequent form. LN invasion was present in 12% of patients, while distant metastases were present in about 3% of patients at diagnosis. According to ATA standards, 29 patients (2.7%) were classified as high-risk. The mean pre-ablation stimulated Tg was 111 μg/l, while the mean Tg at first evaluation was 74 μg/l. The median follow-up period was 5.6 years (**Table 1).**

**Table 1:** General characteristics of the cohort.

Parameter	value or % (±SD) or (IQI)
**Number of patients**	1093
**Median duration of follow up (IQI)**	5.6 years (2.3-9.3)
**Age (years)**	49 (14.8)
**Sex: Female (%)**	78
**Histology: Papillary (%)**	88
**T (%)**	
T1	55.8
T2	19.4
T3	22.1
T4	2.4
**Main lesion’s diameter (cm)**	1.89 (1.4)
**Thyroid capsule invasion (%)**	24.39
**N (%)**	
Nx	72.4
N0	13.1
N1a	8
N1b	4.3
**Number of invaded LN**	4.3 (4.3)
**Extra nodal extension (%)**	43
**Distant metastases (%)**	3.1
**High risk of recurrence (%)**	2.9
**Pre-ablation sTg (μg/l)**	111 (1685)
**Tg (μg/l) at evaluation**	74 (2220)

Data is expressed as patients’ number (%) unless indicated otherwise. Pre-ablation sTg: pre-ablation stimulated thyroglobulin just before RAI ablation; Tg at evaluation: thyroglobulin post-rhTSH at 1^st^ evaluation (6-9 months after RAI ablation).

Univariate analysis found sex, age, histological type, pTNM, initial ATA risk, pre-ablation sTg and Tg at initial evaluation to be predictive factors of long-term survival **(Table 2).** In multivariate analyses, initial ATA high-risk status (HR 21.9; 95% CI: 8.5-56.3), age over 55 years (HR 23.8; 95% CI: 7.5-75.3), and pre-ablation sTg ≥30 μg/l (HR 8.4; 95% CI: 4.6-15.3) significantly impacted 10-year survival in patients treated for DTC. Age over 45 years, follicular subtype and ATA intermediate-risk were also significant, but to a lesser extent **(Table 2).** Only a tendency was observed for male sex and for pT4 stage tumors, while Tg (Tg: measured 72 hours after human recombinant TSH injection used for the first evaluation of RAI efficacy), thyroid capsule invasion, lymph node invasion and extra nodal extension did not affect long-term survival in these patients (**Table 2**).

**Table 2:** Univariate and multivariate analyses determining the predictive factors of ten-year survival in the cohort.

Parameters	10-year survival % patients [95%-CI]	*P*	Multivariate OR (95%-CI)	*p*
**Sex**				
**Female**	94.9 [92.3-96.6]	0.001		
**Male**	86.1 [76.9-92.1]		1.82 (0.96-3.45)	0.28
**Mean age**				
< 45 years	100	<10^-7^		
45-55 years	95.8 [90.4-98.2]		**4.9 (2.7-8.7)**	<10^-7^
55 ≥ years	82.4 [75.4-87.7]		**23.8 (7.5-75.3)**	
**Histological type**				
Papillary	94.8 [92.2-96.6]	0.006		
Follicular	87 [72.8-94.4]		**3.1 (1.6-5.7)**	0.00045
**pT1-T3**	93.5 [90.8-95.5]	<0.0001		
**pT4**	77.8 [57.3-90.2]		1.9 (0.99-3.2)	0.058
**Thyroid capsule invasion**				
Yes	91.1 [84.9-94.9]	<0.01	0.64 (0.3-1.5)	0.32
No	94.6 [91.4-96.7]			
**N**		0.35		
N0-Nx	93.2 [90.1-95.3]			
N1a	93.5 [85.5-97.2]			
N1b	91.8 [78.1-97.3]			
**Extra nodal extension**				
Yes	89.3 [77.1-95.4]	0.26		
No	98.1 [89.9-99.7]			
**Pre-ablation sTg**				
sTg ≤10 μg/l	94.4 [91.1-96.5]	<10^-7^		
sTg 10-29 μg/l	96.2 [90.2-98.6]			
sTg ≥30 μg/l	78 [67.2-86.3]		**8.4 (4.6-15.3)**	<10^-7^
**Tg at evaluation**				
Tg <1 μg/l	98 [89.5-99.7]	<10^-5^		
Tg 1-10 μg/l	94.2 [87.8-97.3]			
Tg >10 μg/l	76.1 [61.1-95.3]		0.76 (0.5-3.1)	0.58
**Initial low risk of recurrence**	95.0 [91.5-97.2]	<10^-7^		
**Initial intermediate risk of recurrence**	92.6 [87.8-95.6]		**4.7 (2.9-7.5)**	<10^-7^
**Initial high risk of recurrence**	51.3 [30.2-71.9]		**21.9 (8.5-56.3)**	

Pre-ablation sTg: pre-ablation stimulated thyroglobulin just before RAI ablation; Tg at evaluation: thyroglobulin post-rhTSH at 1^st^ evaluation (6-9 months after RAI ablation).

### Influence of initial risk stratification on long-term disease-specific survival

**Fig 1:**
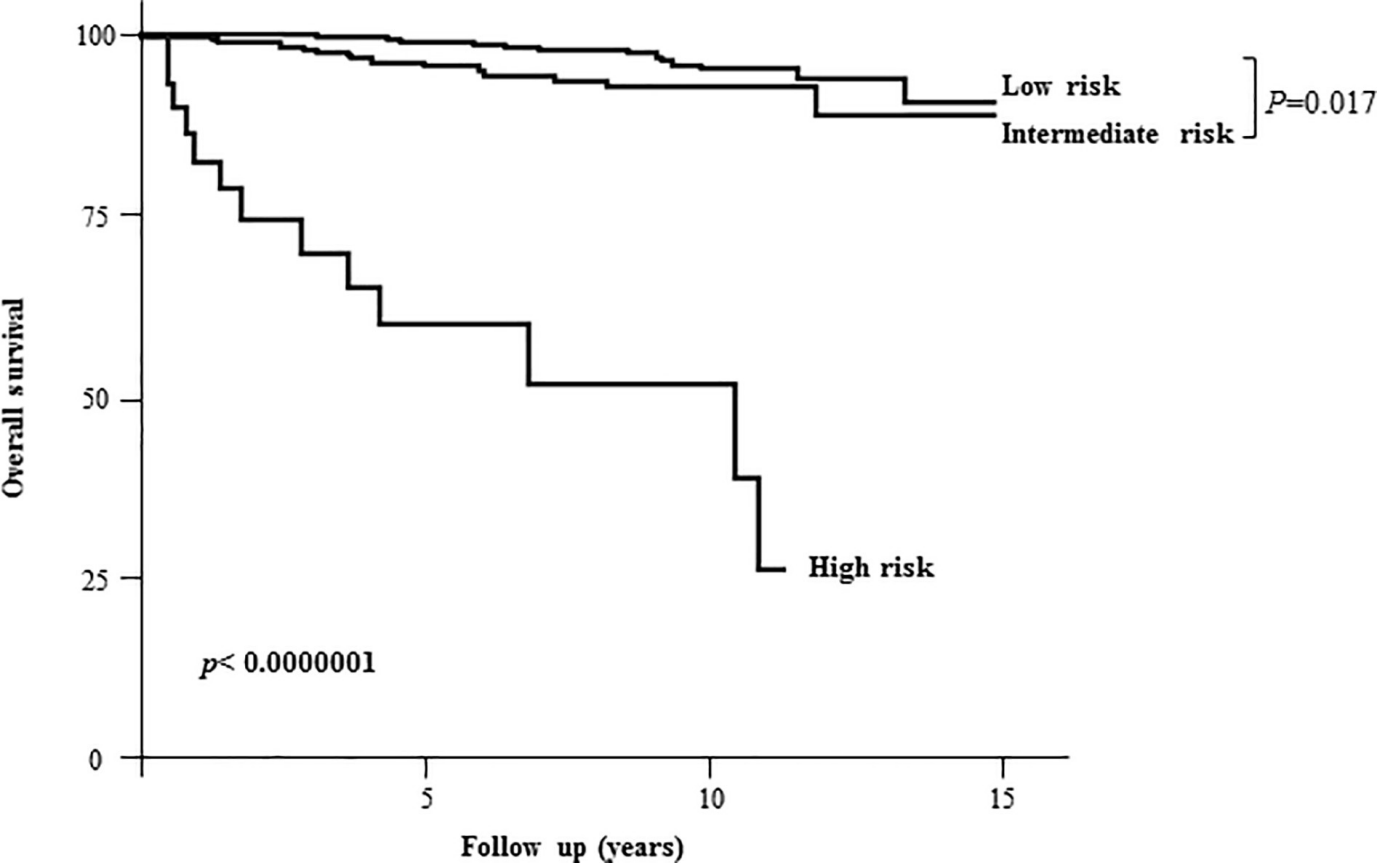
Kaplan Meier analysis of 10-year disease-specific survival according to the initial risk stratification (based on clinical/pathological/biological data during thyroid ablation).

Kaplan Meier long-term survival estimates showed a significant reduction in the overall survival probabilities in patients in the ATA high-risk group (**Fig 1**). We compared patients according to their initial risk stratification, as suggested by the ATA. Patients in the ATA high-risk group (mostly patients with distant metastases, 2.7% of the population) were older (59 years old *vs* 48 and 50 years old; p<0.01), with a lower female ratio (62% vs 83% and 69%; p<10^-5^) compared to the low- and intermediate-risk groups. Furthermore, mean pre-ablation sTg and Tg at evaluation were significantly higher (3539 μg/l and 5747 μg/l respectively) compared to the intermediate-risk (42μg/l and 5μg/l respectively) and low-risk groups (6.5 μg/l and 0.82 μg/l respectively) (p<10^-5^) **(Table 3).** Indeed, in the ATA high-risk group, 59% of the patients had pre-ablation sTg over 30 μg/l (p<10^-7^) and 55% of them had Tg at evaluation higher than 10 μg/l (p<10^-4^). Death rates were 45% in the high-risk group *vs* 2.2% and 5.2% in the low-risk and intermediate-risk groups, respectively **(Table 3).**

**Table 3:** Characteristics of patients classified according to ATA initial risk staging.

Parameters	ATA Low-risk	ATA Intermediate-risk	ATA High-risk	*P*
**No. of patients (%)**	738 (67.5)	326 (29.8)	29 (2.7)	
**Deceased patients**	16 (2.2)	17 (5.2)	13 (44.8)	
**Median duration of follow up [IQI]**	5.6 [2.2-9.2]	5.7 [2.5-9.7]	3.8 [1.2-6.4]	
**Sex**				
Female	611 (83.5)	225 (69)	18 (62)	<10^-5^
Male	121 (16.5)	101 (31)	11 (38)	
**Mean age (years±SD)**	48.5±14.2	49.8±16.7	58.6±18.6	<0.01
**Pre-ablation sTg**				
Mean (μg/l±SD)	6.5±16.7	42.2±179	3539±9723	<10^-5^
sTg ≤10 μg/l	620 (84)	229 (70.3)	7 (24.1)	<10^-7^
sTg 10-30 μg/l	91 (12.4)	49 (15)	5 (17.2)	
sTg ≥30 μg/l	27 (3.6)	48 (14.7)	17 (58.7)	
**Tg at evaluation**				
Mean (μg/l±SD)	0.82±5.4	5.03±41.1	5746.9±11993	<10^-5^
Tg <1 μg/l	586 (90.6)	221 (76.2)	7 (24.1)	<10^-4^
Tg 1-10 μg/l	59 (9.1)	56 (19.3)	6 (20.7)	
Tg >10 μg/l	2 (0.3)	13 (4.5)	16 (55.2)	

Data is expressed as patients’ number (%) unless indicated otherwise. Pre-ablation sTg: pre-ablation stimulated thyroglobulin just before RAI ablation; Tg at evaluation: thyroglobulin post-rhTSH at 1^st^ evaluation (6-9 months after RAI ablation).

### Influence of age and pre-ablation thyroglobulin on long-term disease-specific survival

**Fig 2:**
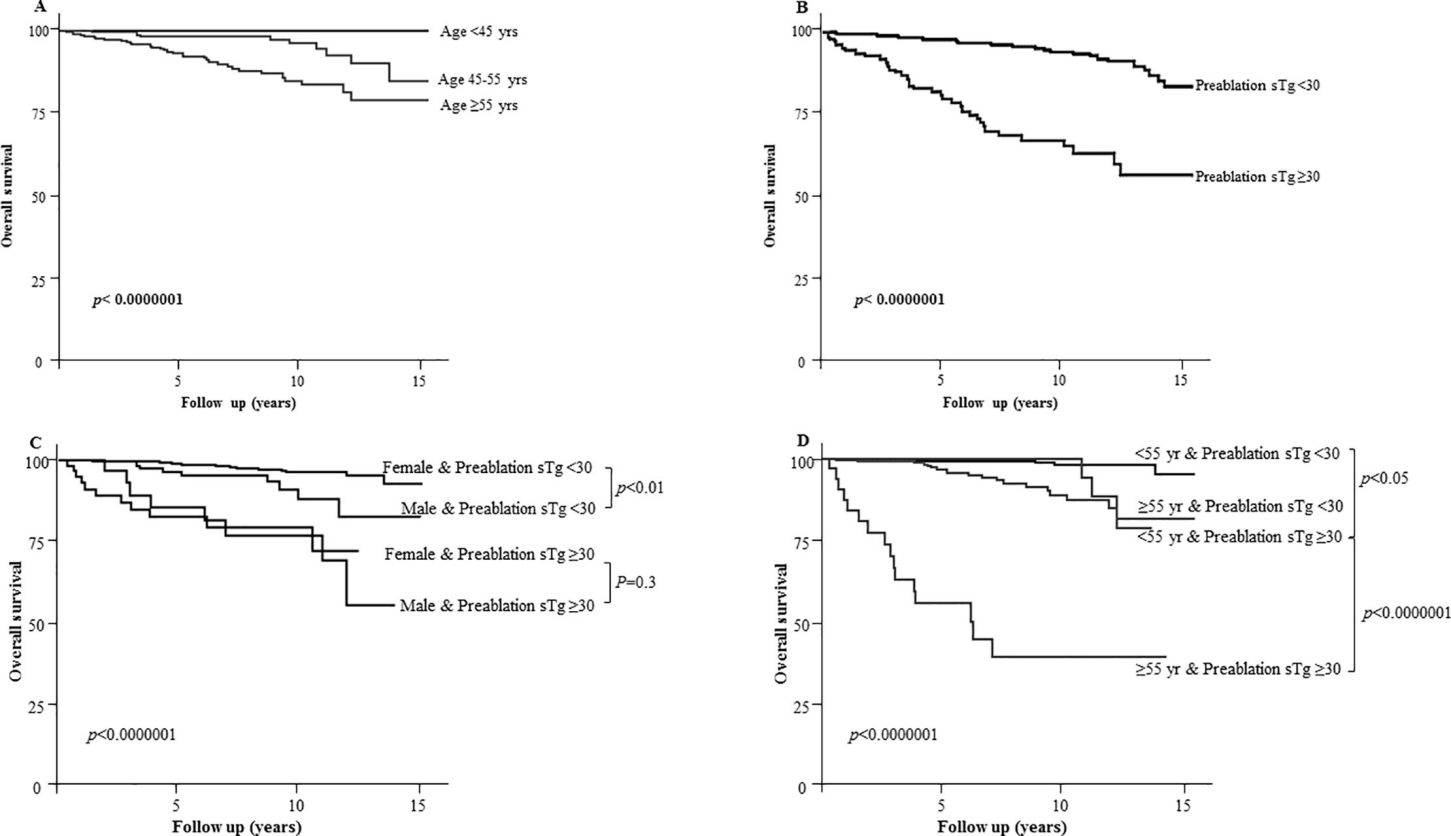
Influence of age and pre-ablation sTg on 10-year disease-specific survival in patients managed for DTC. A- Kaplan Meier survival in patients according to age: >45 years (according to 7^th^ AJCC/TNM system) and ≥55 (according to 8^th^ AJCC/TNM system). B- Kaplan Meier survival in patients with pre-ablation sTg < or ≥30 μg/l. C- Kaplan Meier survival in patients with pre-ablation sTg < or ≥30 μg/l according to sex. Please note that the difference is statistically significant between men and women with pre-ablation sTg <30 μg/l (p = 0.0062). The influence of sex is lost when pre-ablation sTg ≥30 μg/l (p = 0.3). D- Kaplan Meier survival in patients with pre-ablation sTg < or ≥30 μg/l according to age < or ≥55 years. Note the prognosis of patients older than 55 years having pre-ablation sTg ≥30 μg/l.

ATA high-risk classification (**Fig 1**), age over 55 years (**Fig 2A**) and pre-ablation sTg were major determinants of survival in our cohort. To determine a predictive value for pre-ablation stimulated thyroglobulin, an analog of ROC analysis was performed with an optimal cutoff in our population (**S1 Fig**) and validated by Kaplan Meier estimations of survival rates for different cutoff values (**S1 Fig**). Pre-ablation sTg ≥30 μg/l was associated with the lowest survival rates (**Fig 2B**).

Next, we analyzed the influence of pre-ablation sTg on overall survival rates, taking into consideration patients’ sex and age. The difference in survival rates remained significant between men and women when the pre-ablation sTg was <30 μg/l (p<0.01), however, the influence of sex disappeared when the pre-ablation sTg ≥30 μg/l (p = 0.3) (**Fig 2C**). Thus, women with pre-ablation sTg equal or higher than 30μg/l no longer had a prognostic advantage over men.

Survival rates were excellent in patients younger than 55 years old with pre-ablation sTg <30 μg/l (**Fig 2D**). A rapid decline in the survival rate was observed in patients older than 55 years old who had a pre-ablation sTg ≥30 μg/l, with rates lower than 5 years in more than 50% of patients (**Fig 2D**). Consequently, these results highlight three populations according to their age and pre-ablation sTg levels. In the **“excellent survival group”** (age <55 years with pre-ablation sTg <30; 59% of the cohort), 7 (1%) patients died (Median follow up: 5.9 years [IQI: 2.3-9.6]). In the **“moderate survival group”** (age <55 years with pre-ablation sTg ≥30 or ≥55 years with pre-ablation sTg <30; 38.1% of the cohort), 23 patients (5.5%) died (Median follow up: 5.3 years [IQI: 2.2-8.8]). In the **“low survival group”** (age ≥55 years with pre-ablation sTg ≥30; 2.9% of the cohort), 16 patients (51%) died (Median follow up: 3.9 years [IQI: 1.8-7.1]) (**Fig 2D**).

## Discussion

In this study, we determined the main negative factors that influence long-term disease-specific survival in patients with DTC who were treated with thyroidectomy and RAI. According to the ATA’s standards, 3% of patients were classified as having a high-risk of recurrence based on the initial treatment. The ten-year disease-specific survival was lower in patients with a high risk, patients who were older than 55 years, and in patients with high pre-ablation sTg (pre-ablation sTg ≥30 μg/l) levels. A rapid decline in the survival rates was observed in patients older than 55 years who had a pre-ablation sTg ≥30 μg/l. To stratify disease follow-up, we suggest three categories of survival rates to be distinguished apart from sex or TNM staging: excellent (patients <55 years with pre-ablation sTg <30 μg/l), moderate (patients <55 years with pre-ablation sTg ≥30 or ≥55 years with pre-ablation sTg <30), and low survival rate (patients ≥55 years with pre-ablation sTg ≥30 μg/l).

As reported previously in the literature, the ten-year survival of the cohort was excellent [8,13]. The prevalence of distant metastases in our cohort is also identical to what is described in the literature [7]. Indeed, long-term survival is affected by the extent of surgery (lobectomy versus thyroidectomy) and by tumor size (>4 cm) [7,14]. However, the prognosis of patients with DTC seems to be independent of prophylactic lymph node dissection (ipsilateral or bilateral) in clinically node-negative disease [15]. Even in patients with lymph node micro metastases, long-term survival seems unaffected by lymph node dissection [16]. Although these studies give a high consideration for tumor size (> or <4 cm), LN involvement and their influence on the overall survival independently from the type of surgery, they did not take into consideration the disease burden that may be reflected by higher pre-ablation sTg during remnant ablation. Moreover, initial pre-ablation sTg and Tg after human recombinant TSH injection in the first evaluation (Tg) were rarely correlated with overall survival and risk evaluation. Indeed, we have recently demonstrated the importance of pre-ablation sTg in the prediction of long-term remission *versus* disease persistence or recurrence [17]. Matthews et *al*. described patients with a thyroglobulin level of 27.5 μg/l or higher with reduced disease-free survival [18]. Similarly, Podnos et *al*. have described the main predictive factors affecting survival; the presence of distant metastases, large tumor size and lymph node involvement significantly predicted a poor outcome [19,20]. Our results show that primary tumor size (pT) is not a significant factor, while pre-ablation sTg remained highly predictive. Indeed, higher pre-ablation sTg levels reflect an extensive extra thyroidal disease with a significant nodal or metastatic burden, independently from tumor size [20,21]. Higher pre-ablation sTg corresponds either to a very high degree of lymph node involvement and/or to extra nodal extension, which is why lymph node invasion did not affect survival in our cohort [2,8]. Furthermore, higher pre-ablation sTg≥30 μg/l is associated with lower survival rates to the same degree in both men and women.

In our study, age is a significant factor, especially age over 55 years old. Indeed, age above 45 years old was correlated with a reduced survival rate as suggested by many other studies, including the latest ATA references [2,8,9]. With the new staging AJCC/TNM system, the age cut-off was changed to 55 years [22,23]. Interestingly, the 8^th^ edition of the staging system takes into consideration age and TNM. Indeed, patients older than 55 years with pT4 tumors or M1 are considered as stage 4, corresponding to a drastic decline of the survival rates. This system does not take into consideration the pre-ablation sTg levels. Herein, our results are complementary to the staging system based on age and TNM. We suggest to incorporate the pre-ablation stimulated Tg as a complement of the staging system. With pre-ablation sTg cutoff, the survival rates are separated in three categories independent of other confounding factors such as sex or ATA recurrence risk stratification.

This study demonstrates the importance of a very high pre-ablation sTg and older age in survival rate estimations. Pre-ablation sTg is complementary to pTNM classification when predicting survival in patients with DTC, and patients older than 55 years old with a pre-ablation sTg ≥30 μg/l could be differentiated from others. High survival rate group patients are younger than 55 years old with a pre-ablation sTg < 30 μg/l; moderate survival group patients are either young with a pre-ablation sTg ≥30 μg/l or older with a pre-ablation sTg <30 μg/l; the low survival rate group patients have pre-ablation sTg ≥30 μg/l and are older than 55 years old. Low survival rate patients represent 3% of our cohort and have the worst prognosis. They require further attention and aggressiveness in their disease management.

One of the limitations of this study is that patients treated with RAI after rhTSH stimulation were eliminated. Indeed, methods of stimulation and their intensity between thyroid hormone withdrawal and recombinant human TSH differ. Furthermore, the pre-ablation sTg measurement was performed on Day 5 after RAI in the THW group and on Day 3 in rhTSH group. Also, these patients were different; rhTSH was employed mostly in elderly patients with a low risk disease. In addition, usually 30 mCi instead of 100 mCi were administered to the low risk patients. Thus, including such a population would be a source of bias. It would be interesting to evaluate patients with rhTSH stimulation independently to validate such parameters.

Another limitation of this study is the TNM staging system employed by our database (AJCC 2010: 7th edition). We incorporated thyroid capsule invasion (the difference between T2 and T3) and the tumor diameter in order to be well-matched with the 8^th^ TNM edition. The later version was published after the completion of the collection of our results (2017). Besides, some patients of our cohort with low ATA risk (tumor size of 1-4 cm with or without microscopic central compartment nodal extension; T1b-T2, N1a) were probably unnecessarily treated with RAI, if we take in consideration the new ATA recommendations. However, data and recommendations about treating or not these patients remain conflicting [2]. Furthermore, the possible “overtreatment” with RAI of some low risk patients in our study would not affect our results, as the intermediate and high risk groups should be systematically treated with RAI.

Though a continuous update of patients’ data was performed with time (prospectively), this study remains retrospective and non-randomized. Moreover, we highlight that the pre-ablation sTg was measured after THW while Tg was measured after rhTSH injections, however these measurements respect the national and international guidelines of the management of DTC needing RAI treatment [2].

## Conclusion

Our study demonstrates excellent long-term survival rates in DTC patients treated by surgery followed by radioactive iodine ablation. Initial ATA high-risk classification, age over 55 years old and pre-ablation sTg ≥30 μg/l are the main negative factors that influence the ten-year disease specific survival in patients with DTC. A rapid decline in the survival rates is observed in patients older than 55 years old who have a pre-ablation sTg ≥30 μg/l.

We suggest that the cutoff of pre-ablation sTg ≥30 μg/l be integrated in initial survival-risk stratification. Three categories of survival rates could be highlighted: a high survival rate group, consisting of patients who are younger than 55 years with pre-ablation sTg <30 μg/l; a moderate survival rate group, consisting of patients who are either young with pre-ablation sTg ≥30 μg/l or patients older than 55 years with pre-ablation sTg <30 μg/l; and finally, a low survival rate group, consisting of patients who have pre-ablation sTg ≥30 μg/l and are older than 55 years old.

## Supporting information

S1 Fig Determination of pre-ablation sTg cutoff that influence 10-year disease specific survival. A- Exhaustive research for a predictive pre-ablation sTg in our cohort thanks to a ROC analog analysis.
B- Kaplan Meier survival in patients with different pre-ablation sTg levels (<1, 1-5, 5-10, 10-30 and ≥30 μg/l).
(TIF)
