# Supplementary figures and images for: Age, thyroglobulin levels and ATA risk stratification predict 10-year survival rate of differentiated thyroid cancer patients
Source: PLoS One. 2019 Aug 19;14(8):e0221298. doi: 10.1371/journal.pone.0221298 (PMC6699685; doi:10.1371/journal.pone.0221298)

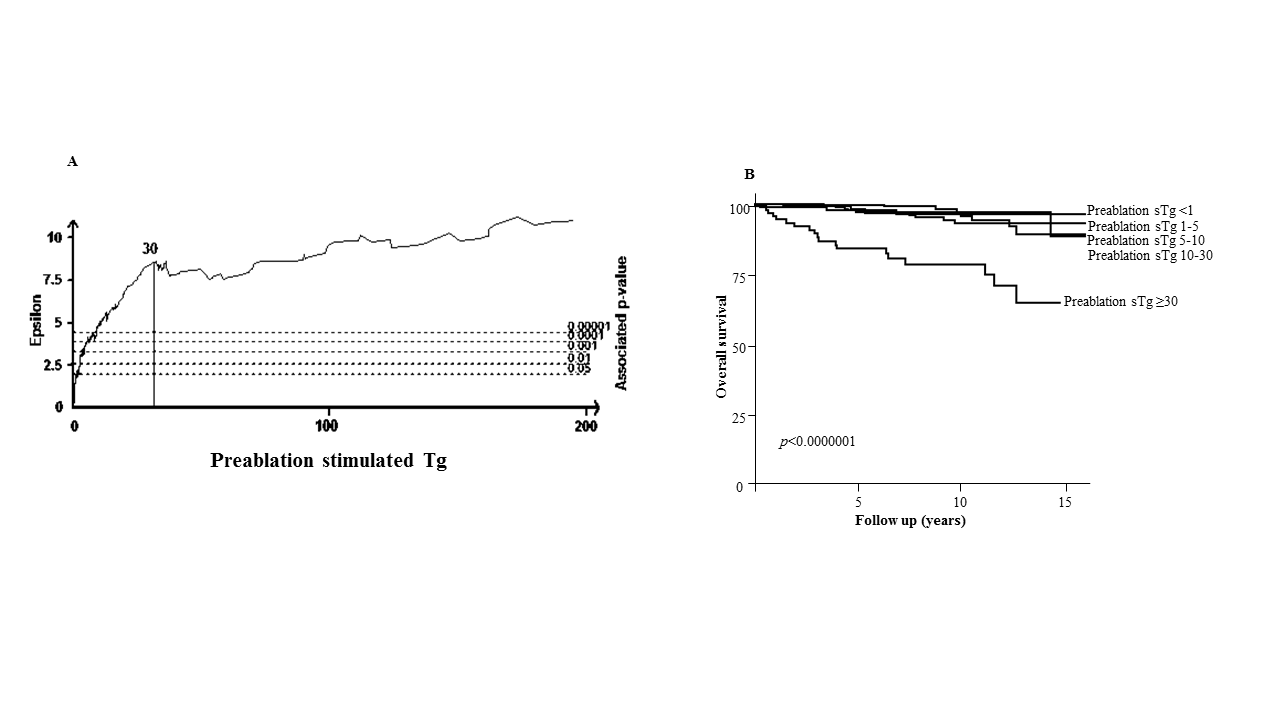

Supplement: S1 Fig — A- Exhaustive research for a predictive pre-ablation sTg in our cohort thanks to a ROC analog analysis. B- Kaplan Meier survival in patients with different pre-ablation sTg levels (<1, 1-5, 5-10, 10-30 and ≥30 μg/l). (TIF) [file pone.0221298.s001.TIF]
